# Effects of Nuclear Genomes on Anther Development in Cytoplasmic Male Sterile Chicories (*Cichorium intybus* L.): Morphological Analysis

**DOI:** 10.1155/2015/529521

**Published:** 2015-03-15

**Authors:** Ildephonse Habarugira, Theo Hendriks, Marie-Christine Quillet, Jean-Louis Hilbert, Caroline Rambaud

**Affiliations:** ^1^UMR 1281, Stress Abiotiques et Différenciation des Végétaux Cultivés, Université Lille 1, Sciences et Technologies, 59655 Villeneuve-d'Ascq, France; ^2^University of Rwanda-Collège of Education, P.O. Box 5039, Kigali, Rwanda; ^3^Laboratoire Evolution Ecologie Paléontologie, Bât SN2, Cité Scientifique, Université Lille 1, Sciences et Technologies, 59655 Villeneuve-d'Ascq, France; ^4^Laboratoire Régional de Recherche en Agroalimentaire et Biotechnologie, Institut Charles Viollette, Bât SN2, Cité Scientifique, Université Lille 1, Sciences et Technologies, 59655 Villeneuve-d'Ascq, France

## Abstract

The *Cichorium intybus* flower development in fertile, cytoplasmic male sterility (CMS 524) and various phenotypes carrying the 524 male sterile cytoplasm was investigated macroscopically and by light microscopy. The development was similar in fertile and in male sterile florets up to meiosis, and then it was affected in anther wall structure and pollen grain development in male sterile floret. In the male sterile plants, the tapetum intrusion after meiosis was less remarkable, the microspores started to abort at vacuolate stage, the connective tissue collapsed, and endothecium failed to expand normally and did not undergo cell wall lignification, which prevented anther opening since the septum and stomium were not disrupted. Crosses undertaken in order to introduce the CMS 524 into two different nuclear backgrounds gave rise to morphologically diversified progenies due to different nuclear-mitochondrial interactions. Macroscopic and cytological investigations showed that pollen-donor plants belonging to Jupiter population had potential capacity to restore fertility while the CC line could be considered as a sterility maintainer.

## 1. Introduction

Cytoplasmic male sterility (CMS), a maternally inherited deficiency in producing viable pollen [1], may appear spontaneously or be induced by inter- or intraspecific crosses or by protoplast fusion. It has been described in over 150 plant species [2] including green bean, beet, carrot, maize, onion, petunia, rice, rye, sunflower, and wheat. In CMS types where cytological analysis has been done, the developmental deviation from the normal development pattern can occur at any stage of anther development, either before, during, or after meiosis [3, 4]. For instance anther development was reported to be affected early in one* Citrus* CMS type in which anther abortion occurred during initiation of anther development [5]. In the sunflower CMS type, named PET1, the pollen is not formed due to premature tapetum degeneration and microspore tetrad disintegration soon after the second meiotic division resulting in sterility [6–9]. In the* Petunia pcf* male sterile flower, the tapetal cells are disorganized before their degeneration and the microspores degenerate in the callose wall, which is not degraded [10]. However, in other CMS systems, anther development may be normal until late stages of pollen development and male sterility is associated with failure of pollen grain maturation like in rice where the pollen development proceeds up to second mitosis [11] or in the S-maize CMS where the pollen fails to accumulate starch [12].

Morphological manifestation of CMS may be limited to anther and pollen development but in some CMS systems developmental changes can be extended to other floral organs morphology and color [13–16]. Moreover, recent studies indicate that CMS-associated genes may affect the expression of floral homeotic genes leading to the transformation of stamens to petals, or stamens to carpels as observed in wheat [17, 18], carrot [19], tobacco [20], or stem mustard [21, 22].

Genetic factors that determine CMS have been proven to be mitochondrially encoded [2, 17, 23] and their effect can be suppressed or counteracted by the products of one or more nuclear genes known as restorer-of-fertility (*Rf*) genes, which restitute the normal flower phenotype and functional pollen [23–25]. The possibility to restore fertility by these genes constitutes a way for understanding the nuclear-cytoplasmic interactions but also makes it possible to exploit the CMS trait in breeding for F1 hybrids production, as already applied in several cultivated species such as sunflower, rapeseed, onion, or sorghum [26]. This process occurs through different ways and in most investigated plant species the* Rf* encode pentatricopeptide repeat (PPR) proteins, which are normally known to be essential for the mitochondrion or chloroplast gene expression [17, 23, 27].

In* Cichorium intybus* hybrid breeding programs are carried out taking advantage of the availability of male sterility. Nuclear male sterility in this species has been observed in a genotype named “Edith” but its use was expensive due to the cost of required propagation of the male sterile parent clones [28]. In such conditions the CMS strategy was a good alternative and CMS plants have been obtained by somatic hybridization between fertile chicory and sunflower protoplasts bearing PET1 CMS [29, 30]. Among the male sterile cytotypes obtained, the so-called “524” and “411” plants [29] were backcrossed to different types of pollen donors in order to transfer the male sterile cytoplasm in new nuclear contexts. The progenies presented a series of flower phenotypes suggesting that the nuclear context could affect the sterility of the cybrids [31]. The pollen donors used belong to the Jupiter (Jup) population and others to the CC line, chosen because the first demonstrated potentiality to restore fertility in some crosses, whereas the second seemed to maintain the male sterility 524 according to macroscopical examination. In some individuals issued from those crosses not only the anther development was altered but also the pistil development was more or less affected. In spite of molecular studies undertaken on the CMS in chicory [29, 31, 32], there is little information about anatomical and cytological changes in flower development, more particularly in the genotypes used for those analyses.

Though pollen development in* C. intybus* has been investigated [33–35], some aspects of earlier anther development as well as anther dehiscence processes in this species are less documented. The early inflorescence and floral ontogeny on* Cichorium* inflorescence has been reported by Harris [36]. This author reported that the sequence of floral initiation on the inflorescence meristem in an acropetal manner was a common feature for Asteraceae species with homogamous heads. Microsporogenesis and gametogenesis have been studied with emphasis on pollen wall development as well as relations between tapetum and the developing microspore and pollen grain [34, 35, 37–39] but there are still conflicting reports about tapetum behavior.

In this paper, we firstly analyzed histomorphological changes during chicory anther development in fertile plants from flower initiation to anthesis and, secondly, by using the normal development as a reference, we investigated alterations in the male sterile 524 and in various phenotypes obtained from crosses between this male sterile plant and two different pollen donors. The influence of the nuclear genome of pollen donor plants on the floral morphology and particularly on anther development in the progenies is discussed.

## 2. Materials and Methods

### 2.1. Plant Material

The plants used for crosses and morphological investigation of* Cichorium intybus* fertile flower development included individuals from the “Jupiter” population and the “CC” line (Table 1).

**Figure 1:**
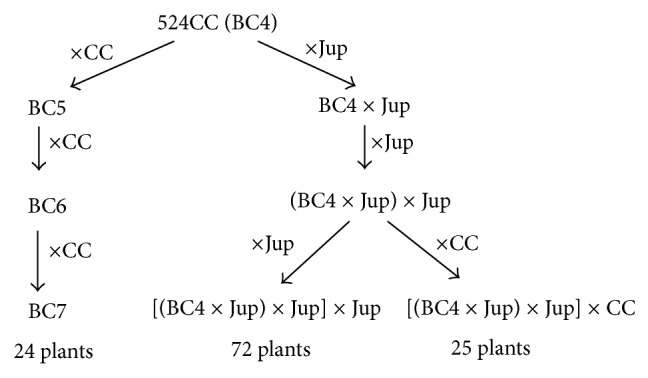
Schematic crossing diagram for maintenance/restoration of cytoplasmic male sterility.

The male sterile cybrid named 524 was obtained by fusion of protoplasts from sunflower male sterile PET1 [40] with those from fertile* C. intybus* L. cv Magdeburg (provided by Ets Florimond Desprez) by Rambaud et al. [29]. A nearly isogenic, alloplasmic male sterile line designated 524CC was created by four successive backcrosses of the cybrid 524 with CC pollen donor line in order to incorporate cytoplasmic male sterility in this line and 524CC (i.e., BC4) was then crossed with pollen donors belonging to CC line and Jupiter (Jup) population (Figure 1). CC was maintained by selfing and four individual plants from Jupiter population were used to produce BC4 × Jup generation. Because of self-incompatibility they were maintained by sister and brother crosses and plants of the progenies were used to produce subsequent backcrosses. In addition, during the three backcrosses, different floral phenotypes (brown or blue anthers) were used as the seed parent, so the 72 3 × Jup progenies represented different families.

Jupiter and CC plants were used as fertile references for the study of the male sterile flower development and plants with different flower phenotypes, which were chosen from the progenies of different crosses described as shown in Figure 1.

Jupiter, CC plants, and their descendants were obtained from seeds and 524CC plants were propagated* in vitro.* After acclimation, they were vernalised (10–12 weeks, 6-7°C) and grown under greenhouse conditions (20 ± 4°C, 80 ± 10% of humidity, and a 16 h photoperiod under ambient light with supplemental lighting when ambient light levels outside were below 3 klux).

### 2.2. Microscopy

Visual examination of the samples was complemented by observations under a Leica LAS stereomicroscope (Leica Microsystems) equipped with a digital camera. For histological analysis, capitular buds of 0.5 to 12 mm in length were collected and categorized into different size classes according to the bud length measured between the base of the receptacle and the top of the bud after removing the involucral bracts. For samples measuring less than 0.5 mm, the length was determined under microscope using the micrometer graduation. The buds were fixed in FAA solution (90 mL ethanol 70%, 5 mL formalin 37%, and 5 mL acetic acid) and dehydrated with an increasing series of ethanol concentrations and a final step in ethanol/butanol (1/1). Capitula were infiltrated with butanol and embedded in glycol methacrylate (Technovit 7100, Heraeus Kulzer). Blocks were sectioned at 4-5 *μ*m (Leica Microtome RM2065), stained with 1% Toluidine Blue O in distilled water, and observed under optical microscope (Olympus BH-2) equipped with a camera (Olympus CAMEDIA c-4000). Sections of at least five capitular buds from the same size class were observed in order to determine the corresponding stage of development.

Sections were stained with periodic acid Schiff (PAS) for starch detection, and for callose detection, sections were incubated in decolorized aniline blue and observed under fluorescence microscope (Olympus BH2-RFCA). To visualize nuclei in the pollen grains the pollen was incubated with DAPI (4,6-diamino-2-phenylindole) and observed under fluorescence microscope. Pollen viability was estimated by using Alexander stain [41].

## 3. Results

### 3.1. Inflorescence and Flower Morphology

**Figure 2:**
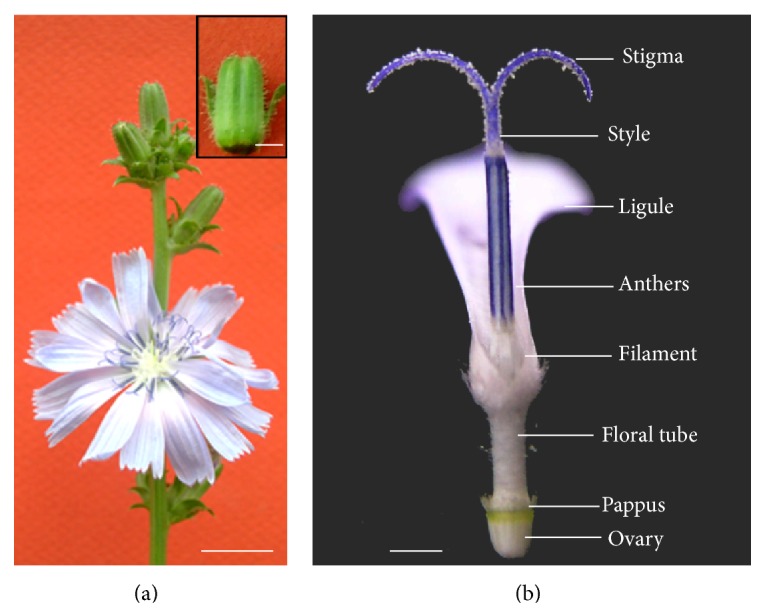
Overview of inflorescence and fertile flower structure. (a) Various stages of capitulum development on a branch with an opened capitulum at anthesis showing ligulate florets. Inset: capitulum with the external bracts partially removed prior to length measurement. (b) The anthers form a column through which the stylar branches and style move upward brushing the pollen grains. The stamens are attached to the ligule and the inferior ovary bears a scaly pappus.* Bars* (a) 1 cm, inset 1 mm, (b) 1 mm.

As in other Asteraceae, the flowers of* C. intybus* are aggregated into heads. At anthesis the capitulum comprises 15–25 hermaphrodite ligulate florets (Figure 2(a)) surrounded by involucral bracts. The anthers are blue and white and form a column surrounding the style (Figure 2(b)). Each of the five stamens is attached to the corolla by the base of its filament. The pistil comprises a unilocular ovary and a style terminating in two (sometimes three) stylar branches (Figure 2(b)). The calyx is reduced to pappus consisting of small scales and the corolla consists of a ligule with five fused petals (Figure 2(b)). The flower development lasts approximately 15 days from flower initiation to anthesis and every capitulum opens only for one day, all the florets being synchronously opened (communication from Theo Hendriks and David Gagneul).

### 3.2. Flower Organs Development in Fertile* Cichorium intybus* Plants

The morphological events during anther development were examined and characterized starting from the appearance of the flower meristem until anthesis. Based on events observed under light microscope, the developmental process in the fertile flower was divided into 16 stages corresponding each to a morphological marker starting from the emergence of the floral meristem and ending with anthesis (Table 2).

### 3.3. Flower Organ Initiation

**Figure 3:**
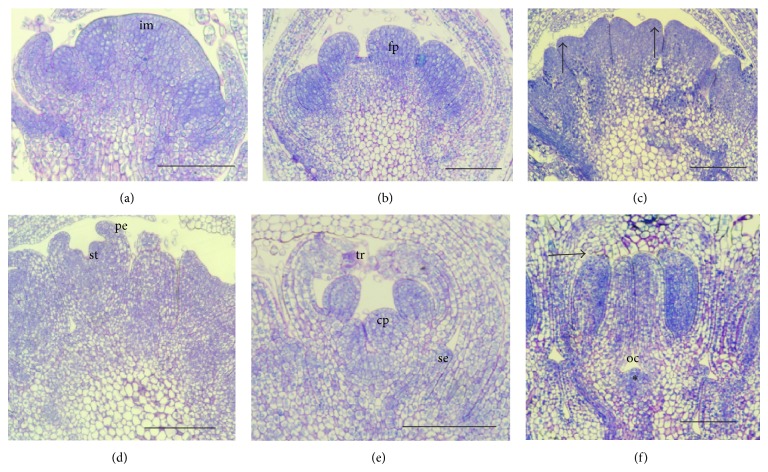
Flower organ initiation (stage 1–5). (a) Inflorescence meristem dome (im) is established. (b) Stage 1: the flower primordia (fp) emerge from the inflorescence meristem. (c) Stage 2: the petals are initiated at the margin of the flower primordium apex (arrows). (d) Stage 3: the stamen primordia (st) are initiated under the petals (pe). (e) Stage 4: the stamens are stalked, sepals (se) are initiated, the carpel lobes (cp) emerge, and trichomes (tr) develop at the top of the petals. (f) Stage 5: the carpel lobes and stamens grow and attain the same height (arrow). Ovary cavity (oc) is formed and ovule is being initiated (∗).* Bars* 100 *μ*m.

From the domed inflorescence meristem (Figure 3(a)) the floret primordia were initiated acropetally and became flattened at their distal part (stage 1, Figure 3(b)). Initiation of the primordia of the floral organs occurred sequentially and began at stage 2 by formation of the primordia of petals at the periphery of the terminal apex (Figure 3(c)). The primordia that will form stamens appeared at stage 3 (Figure 3(d)) and at stage 4 the stamens became stalked (Figure 3(e)) and the gynoecium appeared and rapidly developed two upward bulges which formed the two carpel primordia while the petals developed several trichomes at their apex and bent inward to cover the growing stamens. Sepals were initiated as small protuberances growing slowly and almost simultaneously with the carpel initiation (Figure 3(e)). The region that will give rise to the carpel walls formed the ovary cavity (stage 5, Figure 3(f)) while the two carpel lobes elongated upwards rapidly and fused at their lower regions. At this stage the stamens were of the same height as the carpel lobes (Figure 3(f)) and in cross section, the anther became oval as the filament and anther lobes started to differentiate (Figure 3(f)).

### 3.4. Anther and Pollen Development in Fertile Flower

**Figure 4:**
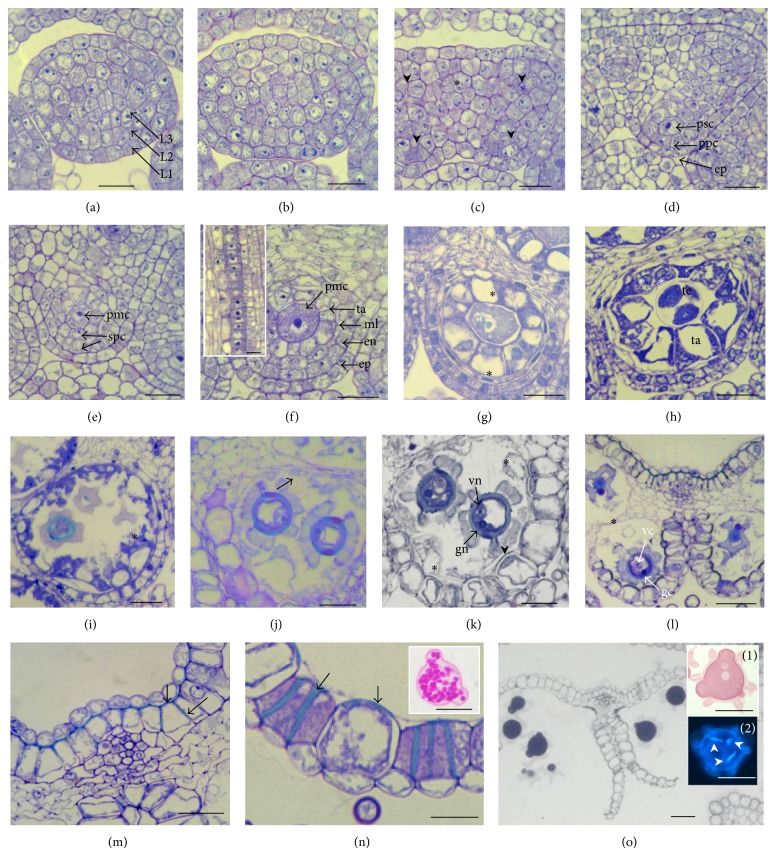
Anther wall and pollen development. (a) Stage 4: round stamen with three germ layers (L1, L2, and L3). (b)-(c) Stage 5: oval shaped anther. The four-locule anther structure established by division of archesporial cells in the four corners (arrow heads). Connectivum differentiation (∗). (d) Stage 6: under epidermis (ep) the primary sporogenous (psc) and primary parietal cells (ppc) are formed. (e) Stage 7: early pollen mother cell (pmc). Two layers form the secondary parietal cells (spc). (f) Stage 8: late pollen mother cells. Four distinguishable anther layers: epidermis, endothecium (en), middle layer (ml), and tapetum (ta). Inset: row of microsporocytes in a longitudinal section at this stage. (g) Stage 9: meiocytes enter meiosis and tapetum enlarges and becomes vacuolated (∗). (h) Stage 10: tetrads of microspores (te). In some tapetal cells two nuclei are visible (∗) (i) Stage 11: release of microspores and tapetal protoplasms intrusion in the locule (∗). (j) Stage 12: uninucleate vacuolate pollen grain. The septum starts to disintegrate (arrow). (k) Stage 13: binucleate pollen grain (gn: generative nucleus and vn: vegetative nucleus). Endothecium expansion and cell wall thickening (arrow heads). The tapetal cell remains are still visible individually in the locule wall (∗). (l) Stage 14: bicellular pollen. (gc: generative cell and vc: vegetative cell). The septum is almost fully disrupted (∗). (m) Magnification of the connectivum region at stage 14 showing U-shaped secondary thickenings (arrows). (n) Magnification of the locule wall showing expanded endothecium cells and their thickened cell walls (arrow heads). Inset: starch grains in the pollen stained by PAS. (o) Stage 15: tricellular pollen. Anther walls have completely opened. Inset 1 shows carbohydrates in the pollen grain stained with PAS and in inset 2 the mature pollen grain with the three nuclei (arrow heads) colored with DAPI.* Bars* 20 *μ*m.

In cross sections, at stage 4, the rounded anther showed the three zones L1, L2, and L3, which will give rise to different anther tissues (Figure 4(a)). Archesporial cells differentiated beneath epidermis (L1) and at stage 5, the anther attained oval shape as the locules, and the connectivum started to differentiate (Figure 4(c)). At stage 6, archesporial cells divided periclinal to give rise to the outer primary parietal layer and the inner one, which formed the sporogenous cells (Figure 4(d)).

By periclinal divisions the primary parietal layer gave rise to two layers, (stage 7, Figure 4(e)) one of which formed the tapetum and the other later divided again to generate the endothecium and the middle layer, when the microspore became more distinguishable at late microsporocyte stage (stage 8, Figure 4(f)). The anther wall finally comprised four cell layers: epidermis (ep), endothecium (en), middle layer (ml), and tapetum (ta) surrounding the longitudinal row of microsporocytes (Figure 4(f), inset). The anther wall development was of dicotyledonous type as the endothecium and the middle layer developed from the same layer. Contrary to the tapetum, which surrounded completely the sporogenous tissue, endothecium and middle layer did not form on the connectivum side of the locule (Figures 4(f) and 4(g)). Through mitosis the primary sporogenous cells (psc) formed secondary sporogenous cells, which developed into pollen mother cells (pmc). These were longitudinally arranged in the anther locule so that in cross section only one microsporocyte could be observed. Before meiosis the meiocytes exhibited a dense cytoplasm (Figure 4(g)) and were surrounded by a thick callosic wall (data not shown). The tapetum cells began to expand just after the four layers were formed at microsporocyte stage (stage 8) and at stage 9 it underwent vacuolation and nuclear divisions which became prominent during meiosis (Figure 4(h)). Meiosis led to microspore tetrads (te) at stage 10 (Figure 4(h)) and the process of cytokinesis was of the simultaneous type.

In the locules of a single anther the development was nearly synchronized but this was not the case for anthers of the same flower. The microspores were released from the callose wall at stage 11; the inner and radial cell walls in the tapetum disintegrated and tapetal protoplasms surrounded the developing microspores in the anther locule (Figure 4(i)) whereas the middle layer started to degenerate. The released microspores had dense cytoplasms and large central nuclei (Figure 4(i)), exine developed except where the three apertures began to form, and the pollen sculpturing continued with the spines formation. The endothecium cells underwent expansion at the onset of the early vacuolate microspore stage (stage 12, Figure 4(j)). After stage 12, endothecium cells were highly vacuolated and bands of lignified secondary thickening were laid down in the cell walls while the tapetum degenerated (Figure 4(k)). The first mitosis in the microspore was unequal thus giving a binucleate pollen grain (Figure 4(k)) with the vegetative cell surrounding the peripheral generative cell (Figure 4(l)). At the binucleate pollen stage (stage 13, Figure 4(k)) some remains of tapetal cells were observed at the edge of the locule and kept their individuality as they did not fuse (Figures 4(j) and 4(k)), indicating a nonperiplasmodial tapetum. It is also at this stage that blue pigmentation was established in the anthers precisely in the epidermal layer of the connectivum.

U-shaped fibrous bands formed in the subepidermal layer of the connectivum and they were oriented in opposite direction to those formed in the endothecium (Figures 4(l), 4(m), and 4(n)). The septum which started to disintegrate at the uninucleate vacuolate microspore stage (Figure 4(j)) disappeared after the bicellular pollen stage, and then the stomium opening followed (stage 14). The second pollen mitosis occurred before anthesis and the pollen grains were released towards the inner side of the anther tube (stage 15, Figure 4(o)). Starch grains accumulated during the pollen maturation phase as revealed by PAS staining (Figure 4(n), insert) but they had disappeared at anthesis (Figure 4(o), inset 1). At anthesis, the second pollen mitosis had occurred and the two sperm cells were spindle-shaped (Figure 4(o), inset 2). The tricellular pollen grains were brushed out of the anther tube, by the elongating pistil moving upward through the anther tube at anthesis. The anther wall consisted only of vacuolated endothecium and epidermis, which shrunk during the stamen senescence later on.

### 3.5. Anther Development in the “524” CMS Plants

**Figure 5:**
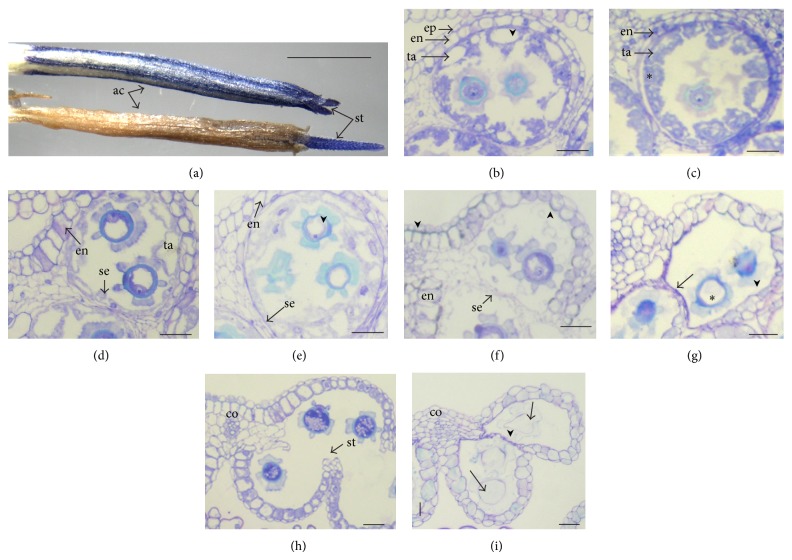
Anther and pollen development in fertile CC line and in 524CC male sterile anther. (a) At anthesis, the fertile anther is blue colored and the sterile is brown and shriveled; the stylar branches (st) arising on the top of the anther column (ac) are normal and blue colored in both cases. ((b), (d), (f), and (h): fertile anthers; (c), (e), (g), and (i): sterile anthers). (b)-(c) Free microspore stage (stage 10). In fertile flower (b) the tapetum has larger vacuoles (arrow heads) and in male sterile flower (c) the vacuoles are numerous but smaller and less significant intrusion of tapetal cells is observed at this stage (∗). (d)-(e) Vacuolated uninucleate pollen grain stage (stage 11). The endothecium (en) expansion is noticeable in (d) not in (e) where the pollen cytoplasm is diffuse (arrow head). (f)-(g) Two-celled pollen grain (stage 13), (f) connectivum and endothecium cell undergo wall lignification (arrow heads), (g) absence of cell wall lignification in the connectivum and endothecium, pollen abortion (∗), shrinkage of endothecium (arrow head) and septum (se) not opened (arrow). (h) The connectivum (co) and endothecium are lignified and the anther is dehiscent. (i) The septum and stomium (st) are unopened (arrow head). The pollen grain is degenerated (arrows), the connectivum is not lignified, and the anther is indehiscent.* Bars* 2 mm in (a), 20 *μ*m in (b)–(i).

At anthesis, the flower of the male sterile line 524CC was distinguishable from the fertile one by its shriveled and brown-colored anthers (Figure 5(a)) and the absence of pollen grains on the style hairs. Stereomicroscopic observations revealed that pollen grains were aborted; the connectivum and the filament were brown conferring this color to the whole stamen. Anther development was similar in the male sterile 524CC and the fertile CC or Jup plants up to meiosis (stage 9). The first visible deviation was the reduced vacuolation of the tapetum (ta) when the tetrads of microspores were formed and its intrusion in the locule space, which was less conspicuous than in the fertile anther (Figures 5(b) and 5(c)). After the microspores were released (stage 11), they exhibited a less dense cytoplasm and nucleus and a large vacuole formed before they degenerated (Figure 5(e)).

In very few pollen grains of 524CC, the first mitosis occurred (stage 13) just before their degeneration. Contrary to the fertile anther, no starch accumulation was observed at any stage of pollen development of the male sterile 524CC and at anthesis most of the pollen grains were shrunken without any observable protoplasm and with abnormally sculptured exine (Figures 5(g) and 5(i)). When in the anther of the fertile plant the pollen was binucleate (stage 13), anthocyanin pigments were observed in the epidermis whereas no blue color was observed in the 524CC male sterile plant anther. Later on, the pollen color in the male sterile 524CC changed from white to brown and this color was extended to the connectivum until anthesis. Moreover, epidermis (ep) and endothecium (en) cells exhibited shriveled shapes and no lignified thickenings were observed in the endothecium cell walls or in the subepidermis of the connectivum (co) (Figures 5(g) and 5(i)). The septum (se) and stomium (st) between adjacent locules which disappeared at the bicellular pollen grain in the fertile flower (Figure 5(h)) shrunk in 524CC anther but were not completely dissolved at anthesis, keeping the locule unopened and shriveled (Figure 5(i)).

### 3.6. Variety of Flower Phenotypes Resulting from Various Crosses

To introduce the CMS into the CC and Jupiter nuclear context, the 524CC male sterile plant was crossed with plants from the Jupiter population and CC line. The progeny exhibited variable flower phenotypes, but for any given progeny plant the floral phenotype was consistent and could be categorized into five classes, and the respective frequencies of their occurrence are shown in Table 3.

**Figure 6:**
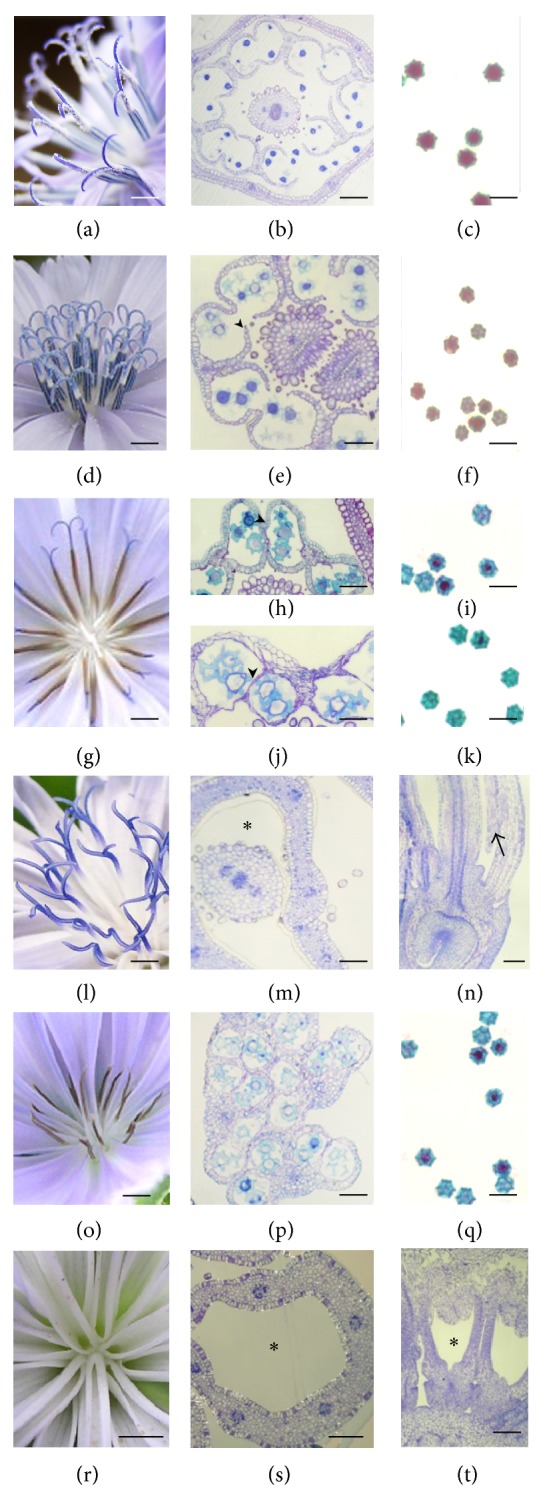
Morphology of flowers and pollen viability in the parents and progenies of crosses with CC and Jupiter pollen donors. (a)–(c) Male fertile flowers: the anthers are blue and the stigma brushing hairs collect pollen grains at anthesis (a). Anther dehisces normally (b) and pollen grains are viable (c). (d)–(f) Class 1: the anthers are blue and the pollen grains collected can be seen on the stylar branches (d). Anthers are dehiscent in (e) (arrow head) but pollen viability is reduced (f). (g)–(k) Class 2: the anthers are brown; the pollen grains are not collected as it can be noticed on the stylar branches and the style. In subclass 2a ((h)-(i)) the septum and stomium are disrupted but the opening of locules is not well achieved (arrow head in (h)) and most of pollen grains are not viable (i). In subclass 2b ((j)-(k)) the anthers remain indehiscent and the locule wall is shrunken (arrow head), no lignification of endothecium and connectivum (j) and the pollen grains are not viable (k). (l)–(n) Class 3: the pistil morphology is normal (l), cross section in the mature floret shows that no stamen develops (∗ in (m)), and longitudinal sections of some florets show some stamen initiation but no differentiation (arrow in (n)). (o)–(q) Class 4: no style is observed (o). The anthers are indehiscent and stacked together (p). Nonviable pollen is shown in (q). (r)–(t) Class 5: no anther and style emerge in the corolla tube (∗ in (r) and (s)). Longitudinal section of young floret buds shows that stamens and carpels are not initiated (∗ in (t)).* Bars* (a), (d), (g), (l), (o), and (r) 2 mm; (b), (e), (h), (j), (m), (n), (p), (s), and (t) 100 *μ*m; (c), (f), (i), (k), and (q) 50 *μ*m.

No segregation of the floral morphology was observed in the progeny after the three backcrosses with CC as all the 24 obtained plants exhibited the brown anther phenotype whereas segregation was observed in the progeny when Jup was used as pollen donor for three successive crosses. Out of 72 plants obtained, 27 and 36 plants exhibited blue anther and brown anther phenotypes, respectively, with additional severely altered phenotypes. When crossed twice with Jupiter and subsequently with CC, segregation of floral morphology was also observed but most of the descendants (22 out of 25) had brown anther phenotype. Besides information provided by visual examination, histological observations allowed characterizing the anther wall structure, pollen development, pollen viability by Alexander staining [41], and pistil development (Figure 6).

*Class 1*. The “*blue anther*” class which was macroscopically similar to the fertile phenotype (Figures 6(a) and 6(b)) exhibited blue anthers and pollen grains were more or less present on the stigma and style at anthesis (Figure 6(d)), but viable pollen grains were often less abundant in the anther than in the normal flower (Figures 6(c) and 6(f)) and in some flowers or capitula, the blue color of the anthers was not stable and tended to turn brown. The stamen and pistil development proceeded as in the fertile flower and, at anthesis, dehiscence appeared normal (Figure 6(e)). 

*Class 2*. The “*brown anther*” class. Like in the class 1, the pistil development was normal. The brown color observed at anthesis was due to aborted pollen grains and the connectivum (Figure 6(g)). At histological level, this class could be divided into two subclasses referring to the structure of the anther wall. In subclass 2a, endothecium cell walls were lignified but even though the dehiscence process occurred, the brushing hairs collected no or very few pollen grains at anthesis. Most of the pollen grains aborted and very few exhibited viability (Figures 6(h) and 6(i)). In subclass 2b the anther wall was exactly identical to that of the male sterile 524CC, that is, no lignification in the endothecium cell walls and in the connectivum which appeared shrunken. The anther is indehiscent and the pollen grains aborted and are nonviable (Figures 6(j) and 6(k)). 

*Class 3*. The “*anther absent*” class exhibited flowers in which only the pistil developed normally (Figures 6(l) and 6(m)). However, in some mature florets rudimentary anther structures could be seen in longitudinal sections at the base of the ligule (Figure 6(n)). In this type of anthers, one or two locules were observed and that did not exhibit well-differentiated walls; the microspores (when present) aborted before first pollen mitosis.

*Class 4*. The “*brown anther*,* style absent*” class (Figures 6(o) and 6(p)). The style was absent or rudimentary and in some florets the ovule was initiated but its development was arrested very early, meaning that the carpel development was severely affected. The endothecium cell walls were not thickened and the anther remained indehiscent, the number of locules was variable from 2 to 4, pollen abortion started after meiosis, and the pollen grains were nonviable at anthesis (Figure 6(q)). 

*Class 5*. The “*anther absent, style absent*” class exhibited the most altered flower phenotype because no structure related to stamen or pistil was observed when the capitulum opened (Figures 6(r)–6(t)) even though the ligule exhibited the same morphological features as in the normal flower. Longitudinal sections in some young capitular buds showed stamen and pistil primordia but they did neither elongate nor differentiate.

## 4. Discussion

In this study the fertile flower development in* C. intybus* was analyzed to allow investigation about morphological alterations in the “524” male sterile line and in the progeny of crosses between this male sterile line and different pollen donor plants. Flower development was divided into 16 stages from the flower initiation to anthesis according to morphological landmarks and the sequence of flower organ initiation (corolla-stamens-gynoecium-pappus) is in accordance with Harris [36].

Anther wall differentiation was according to the dicotyledoneous type [42] since the outer secondary parietal cell layer divisions gave the endothecium, one middle layer, and the inner functioning as tapetum. Due to its considerable physiological importance the tapetum layer attracted attention of several authors [34, 42, 43], and its variation among species has been used to attempt establishing phylogeny of tapetum types among* Embryophyta* [37]. In the Asteraceae family the plasmodial (syn. invasive) tapetum is a common feature [38] even though this type of tapetum is rare in dicots. Pacini and Keijzer [34] have characterized the* C. intybus* as invasive but according to these authors no periplasmodium was formed because the cells did not fuse, whereas Varotto et al. [35] and Chehregani et al. [39] noticed that the tapetal cells fused. According to our observations a part of the cytoplasms remained parietally localized keeping individuality of respective cells, just before the tapetum is completely degenerated. Our results on tapetum behaviour are in agreement with Pacini and Keijzer [34] as individual cells remains are still visible in the locule wall. Recently the occurrence of noninvasive tapetal cells in Asteraceae has been reported in* Carthamus tinctorius* [44], where a small population of tapetal cells is formed as a result of periclinal divisions in the tapetum but does not intrude in the locule. However, such divisions have not been observed in* C. intybus,* suggesting that the parietal cell structures observed correspond rather to remains of the single tapetal cell population than to an additional cell population.

The description of flower development in fertile plants provides a reference for understanding deviations that occur in the male sterile lines. Comparison of anther development in the fertile and “524” male sterile plants allowed us to identify the main morphological alterations that led to male sterility. A combination of alterations in the male sterile 524CC includes the limited tapetum intrusion, pollen degeneration after the free microspore stage, absence of lignification in the anther wall and in the connectivum, absence of anther dehiscence, absence of anthocyanin pigments, and premature shrinkage of the anther.

Anther development follows the same pattern in the fertile and male sterile 524CC up to meiosis when in the latter the tapetum cells were less vacuolated before microspore degeneration, which becomes visible at the vacuolate pollen stage. Tapetum vacuolation was reported in* Arabidopsis thaliana* to be a prominent feature during meiosis before its drastic reduction when the microspores are released from callose walls [45] and these vacuoles were supposed to store enzymes required for callose digestion allowing the microspore release in the anther locule. In the 524-CMS plants, despite the absence of large vacuoles in the tapetum cells during meiosis and the limited intrusion of the tapetum cell protoplasms the microspores are normally released (which suggests that the callose is degraded) but further development is so altered that we can hypothesize a defect of tapetum in its contribution in microspore nutrition and maturation.

Usually, the late anther development stages consist of preparation of anthesis and pollen maturation for a successful pollen presentation when cells of the connectivum between the locules undergo lyses of their middle lamella and degenerate, leading to fusion of the locules and anther dehiscence [46]. All these processes are affected in the 524-CMS suggesting that required enzymatic action for fusion of the locules is not well accomplished in addition to absence of endothecium cell wall thickenings which are reported to be essential in generating shearing forces required for stomium breakage in* Gasteria verrucosa* and* Lilium hybrida* [22, 47–49].

The 524-CMS plants do not exhibit the same anther phenotype as sunflower PET1-CMS from which it was obtained through fusion of protoplasts. The PET1 male sterile line is characterized by disorganization and early degradation of the tapetum, degradation of microspores by the tetrad stage [7], and enlargement of the middle layer [8]. Smart et al. [7] reported that the PET1 phenotype might be related to expression of* orf522* during early stages of the meiocytes leading to their abortion. Considering that in the 524-CMS the microspores are normally released in the locule and the tapetum disintegrating lately compared to PET1-CMS, the cause of the developmental deviation in the two male sterilities is different. This is in accordance with the results of mitochondrial DNA structure analysis, which demonstrated that* orf522* was absent in the 524-CMS genotype [31] suggesting that PET1-CMS and 524-CMS do not share the same molecular mechanism. The anther color in the male sterile “524” flower is brown contrary to the fertile anther which is blue colored. It seems that the 524-CMS expression affects accumulation of pigments in the anther but the relationship between the lack of anthocyanin pigments and this cytoplasmic male sterility is unclear. In fertile florets, the blue color in the connectivum epidermis appears at stage 13, that is, when the pollen is bicellular and after cell wall lignification in the endothecium and in the connectivum subepidermal layer, but in the male sterile 524, these events do not occur. Anthocyanins and lignins biosynthesis pathways share initial reactions that provide phenylpropanoid units by which those secondary metabolites are built. Therefore, absence of anthocyanins in the connectivum epidermis as well as lignification in connectivum and endothecium tissues of the male sterile anther may be a result of inhibition of a key step of the phenylpropanoid pathway. However in the male sterile 524, this inhibition is restricted to anthers as the pigmentation is not affected in other flower parts like pistil and petals, which develop normally and exhibit anthocyanin pigments similar to the fertile flower.

The progeny of crosses involving Jupiter pollen donors as shown in Table 3 demonstrate a high diversity of floral phenotypes, which is probably due to nuclear-cytoplasmic interactions. Given that the backcrosses between the cybrid 524 and CC line plants always produced male sterile progeny exhibiting homogenous flower morphology, the cytoplasm 524 was stable in the CC nuclear context which can consequently be considered as a maintainer line. Contrary to 524, another cybrid, named 411, produced more variable flower phenotypes when introduced in the CC line background, suggesting that the CMS 524 was more stable than CMS 411 in which instability of the mitochondrial genome has also been observed [31, 32]. On the other hand, considering the flower morphology in the progeny obtained with Jupiter pollen donor, total or partial pollen viability and tendency to form blue colored anther and to undergo a normal dehiscence process show involvement of a restoration mechanism. As four plants of the Jupiter population and their progenies had been used for the backcrosses, probably a genetic diversity within Jup plants persisted and accounted in part for the phenotypic diversity of the backcross progenies. To elucidate the genetic control of the restoration of fertility, larger progenies from crosses with individual Jupiter plants are needed.

Even if dehiscence occurred in the “*brown anther*” subclass 2a, very few pollen grains, brown colored, were collected by the brushing hairs at anthesis possibly because the degenerated pollen grains appeared to adhere to each other and to anther walls so that their free mobility was hindered when the style elongated. However, the opening of the anther in this class can be considered as a step in restoration process even if most of the pollen grains are nonviable in this phenotype. The variation of pollen grain viability, anther dehiscence, and color in the progeny from Jupiter and the male sterile 524 suggests that the fertility restoration is inherited as a quantitative trait.

In natural gynodioecious populations the occurrence of intermediate phenotypes in terms of pollen production and viability is usually interpreted as a result of involvement of at least two loci or codominant alleles in the process of restoration [50–52]; the occurrence of a fertility restoration mechanism for artificially created male sterility by fusion of protoplasts is striking and it is of great interest to investigate the origin and distribution of the corresponding restoration alleles in the* C. intybus* species. How restoration alleles can be already present in Jupiter population before the mitochondrial genome modification happened through somatic hybridization in* C. intybus* species remains unclear, unless at least one of the following conditions is considered: (1) restoration alleles naturally exist and are used to restore fertility in similar cytotypes that occur in noninvestigated* C. intybus* populations; (2) during* C. intybus* evolution ancient CMS-associated genes were naturally present in some populations but have not been maintained due to fitness disadvantage of the females whereas restorer genes have been fixed in some genotypes [51, 53]; (3) when a mitochondrial function is altered by the CMS condition, certain molecules could be diverted from their normal function and become recruited in the process of fertility restoration. Such a mechanism may, for instance, involve the so-called pentatricopeptide repeat (PPR) proteins, which are known to be essential for normal mitochondria gene expression [17, 23, 27]. Their role in fertility restoration was reported, for example, in* Petunia* [54],* Sorghum bicolor* [55],* Oryza sativa* [56],* Raphanus sativus* [57, 58], and* Mimulus* [59] acting by suppressing the cytoplasmic dysfunction probably by binding to specific transcripts [60]. In the T cytoplasm-induced male sterility of maize, fertility can be restored by an aldehyde dehydrogenase protein [61] and recently it was shown that in rice, fertility could be restored by a mitochondrial glycine-rich protein [62], which is indicative for the diversity of the fertility restoration mechanisms.

To understand the inheritance of the fertility restoration in the CMS-524 system, a QTL analysis is needed taking advantage of the availability of a chicory genetic map [63]. Then, the next step will be analysing the progeny of crosses involving CC plants carrying the 524-cytoplasm and Jupiter and determining how the fertility restoration inheritance is controlled, that is, identifying the alleles of interest and relationships between them.

In conclusion, the information provided in this study on flower development will be useful for analyzing expression of genes involved in the flower development, in general, and in the male sterilities occurring in* C. intybus* in particular. Moreover, this study constitutes a starting point of the analysis of fertility restoration in the 524-CMS, which may contribute to the understanding of CMS in chicory.

## Figures and Tables

**Table 1:** Parents used for morphological analysis and for different crosses.

Plant	Description	Cytoplasm
CC	Fertile and rapid-cycling line (INRA Versailles). Self-compatible	Normal
524CC	Male sterile line obtained after four backcrosses between the cybrid 524 and the CC line	Male sterile
Jupiter	Fertile genotypes from Jupiter population which belongs to the named “Pain de Sucre.” Self-incompatible	Normal

**Table 2:** Development of male gametophytes in relation to their size and histology.

Bud length (mm)	Stage	Petal, sepals, and stamens
<0.2	1	Flower primordia emergence

0.2–0.3^*^	2	Petal primordia emergence

0.3–0.4^*^	3	Stamen primordia emergence, petals bending

0.5	4	Stalked and rounded stamen, trichomes differentiation on petals, pappus primordia initiation

0.6–0.7	5	Oval shaped stamen in cross section, differentiation into anther and filament, differentiation of archesporial tissue

0.8–1	6	Four-lobed anther, filament elongation and vascularization of anther, formation of sporogenous cells and one parietal cell layer

1.2–1.4	7	Appearance of early pollen mother cells (pmc) and second parietal cell layer

1.5–2	8	Four distinguishable anther layers, about 50 pmc longitudinally aligned in each locule, enlargement and vacuolation of tapetum

2–2.5	9	Callose deposition around pmc, meiosis

2.5–3	10	Tetrads of microspores, crushing of middle layer by the enlarging tapetum, nuclear division in tapetum cells

3-4	11	Degradation of callose, release of microspores, disappearance of middle layer, tapetal cell intrusion in the locule

4-5	12	Vacuolate microspore, expansion of endothecium cells, start of septum disintegration

6	13	First pollen mitosis, start of starch accumulation in the pollen grain, secondary thickenings of endothecium cell wall, anthocyanin biosynthesis in the anther

7	14	Two-celled pollen grain, complete degeneration of tapetum

8-9	15	Tricellular pollen grain, completion of septum disintegration, stomium opening, release of pollen grains in the locule

10–12	16	Anthesis, rapid elongation of the floral tube, pollen collection by the style brushing hairs

^*^The bud length was measured under microscope.

**Table 3:** Frequency of different phenotypes issued from crosses with Jupiter and CC pollen donors.

Cross combinations		Floral phenotypes in the progenies					
♀	♂	Blue anther Class 1	Brown anther Class 2	Anther absent Class 3	Brown anther, style absent Class 4	Anther and style absent Class 5	Total
524CC	3 × CC	0	24^+^ (3)	0	0	0	24
	3 × Jup	27 (6)	37^*^ (2)	2 (2)	5 (1)	1 (1)	72
	2 × Jup; 1 × CC	1	22^*^ (10)	0	1 (1)	1 (1)	25

^+^belongs to subclass 2b and ^*^belongs to subclass 2a after histological analysis. ( ) indicates the number of plants examined under microscope as described below and illustrated in Figure 5.
